# 1,1,4,4-Tetra­methyl­piperazinediium dibromide

**DOI:** 10.1107/S1600536809045139

**Published:** 2009-10-31

**Authors:** Minna Kärnä, Manu Lahtinen, Jussi Valkonen

**Affiliations:** a University of Jyväskylä, Department of Chemistry, PO Box 35, FIN-40014 JY, Finland

## Abstract

A small quantity of the title compound, C_8_H_20_N_2_^2+^·2Br^−^, was formed as a by-product in a reaction between a diamine and an alkyl bromide. The asymmetric unit contains half of a centrosymmetric dication and a bromide anion. In the crystal, weak inter­molecular C—H⋯Br hydrogen bonds consolidate the crystal packing.

## Related literature

For a possible synthetic route, see Creighton & Taylor (1987 ▶). For related structures, see; Linden *et al.* (1999 ▶, 2002 ▶); Guo *et al.* (2007 ▶).
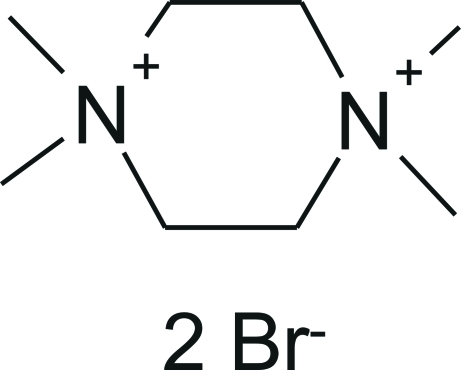


## Experimental

### 

#### Crystal data

C_8_H_20_N_2_^2+^·2Br^−^
*M* *_r_* = 304.08
Monoclinic, 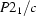

*a* = 5.8769 (12) Å
*b* = 8.4584 (17) Å
*c* = 11.200 (2) Å
β = 92.79 (3)°
*V* = 556.07 (19) Å^3^
*Z* = 2
Mo *K*α radiation
μ = 7.25 mm^−1^
*T* = 123 K
0.24 × 0.16 × 0.16 mm

#### Data collection

Bruker Kappa APEXII diffractometer
Absorption correction: multi-scan (*SADABS*; Sheldrick, 2004 ▶) *T*_min_ = 0.296, *T*_max_ = 0.390
5032 measured reflections
1370 independent reflections
1223 reflections with *I* > 2σ(*I*)
*R*_int_ = 0.032

#### Refinement

*R*[*F* ^2^ > 2σ(*F* ^2^)] = 0.025
*wR*(*F* ^2^) = 0.061
*S* = 1.11
1370 reflections
95 parameters
All H-atom parameters refined
Δρ_max_ = 0.38 e Å^−3^
Δρ_min_ = −0.70 e Å^−3^

### 

Data collection: *COLLECT* (Nonius, 1999 ▶); cell refinement: *DENZO-SMN* (Otwinowski & Minor, 1997 ▶; Otwinowski *et al.*, 2003 ▶); data reduction: *DENZO-SMN*; program(s) used to solve structure: *SHELXS97* (Sheldrick, 2008 ▶); program(s) used to refine structure: *SHELXL97* (Sheldrick, 2008 ▶); molecular graphics: *Mercury* (Macrae *et al.*, 2006 ▶); software used to prepare material for publication: *SHELXL97*.

## Supplementary Material

Crystal structure: contains datablocks I, global. DOI: 10.1107/S1600536809045139/cv2642sup1.cif

Structure factors: contains datablocks I. DOI: 10.1107/S1600536809045139/cv2642Isup2.hkl

Additional supplementary materials:  crystallographic information; 3D view; checkCIF report

## Figures and Tables

**Table 1:** Hydrogen-bond geometry (Å, °)

*D*—H⋯*A*	*D*—H	H⋯*A*	*D*⋯*A*	*D*—H⋯*A*
C2—H2*A*⋯Br1^i^	0.96 (3)	2.88 (4)	3.816 (4)	165 (2)
C2—H2*B*⋯Br1	0.93 (3)	2.92 (4)	3.820 (4)	163 (2)
C2—H2*C*⋯Br1^ii^	0.98 (3)	2.83 (4)	3.770 (3)	161 (2)
C3—H3*B*⋯Br1^iii^	1.00 (3)	2.84 (3)	3.806 (4)	163 (2)
C4—H4*A*⋯Br1^iv^	0.93 (3)	2.92 (2)	3.566 (2)	127 (2)
C4—H4*B*⋯Br1^ii^	0.97 (3)	2.86 (5)	3.787 (2)	159 (2)

Symmetry codes: (i) 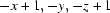; (ii) 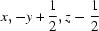; (iii) 
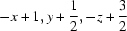
; (iv) 
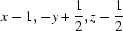
.

**Fig. 1.**
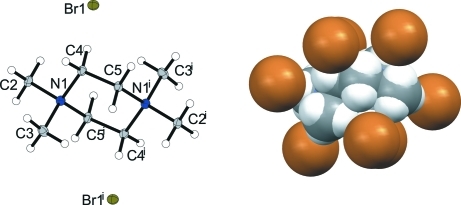
Left: The molecular structure of (I) showing 50% probability displacement ellipsoids and the atomic numbering [symmetry code: (i) -x + 1, y + 1/2, -z + 3/2]. Right: Spacefill presentation of location of eight bromides around a single dication. Six of the anions belong to the neighboring ion pairs.

## supplementary crystallographic information

### Comment

Low quantity of the title compound (Fig. 1) was formed as a byproduct in a synthesis between a tetramethylethylenediamine (TMEDA) and ethoxyethylbromide. Most probably residues of dibromoethane existed as an impurity on either of the starting materials as it is known that piperazinium can be formed by reacting TMEDA and 1,2-dibromoethane. The compound has been recrystallized from acetonitrile/methanol solvent and its crystal structure is reported here.

The asymmetric unit consists of one anion and half a cation. The C—H···Br distances vary from 2.826 (30) to 2.924 (20) Å. In the crystal, cations are packed columnary along *a* axis forming at the same time layers along *b* axis. The bromide anions are analogously packed between the cation layers. The structure is stabilized by weak intermolecular C—H···Br interactions. Cation conformation of this compound is similar to those previously reported tetraiodidocadmate and pentabromothallate salts.

### Experimental

The compound was a byproduct from a reaction between tetramethylenediamine and ethoxyethylbromide. Few crystals suitable for a single-crystal structure determination recrystallized from an acetonitrile-methanol solution.

### Refinement

All H atoms were located from the difference map and refined isotropically.

### Crystal data

**Table:**

C_8_H_20_N_2_^2+^·2Br^−^	*F*(000) = 304
*M**_r_* = 304.08	*D*_x_ = 1.816 Mg m^−^^3^
Monoclinic, *P*2_1_/*c*	Mo *K*α radiation, λ = 0.71073 Å
*a* = 5.8769 (12) Å	Cell parameters from 3094 reflections
*b* = 8.4584 (17) Å	θ = 0.4–28.3°
*c* = 11.200 (2) Å	µ = 7.25 mm^−^^1^
β = 92.79 (3)°	*T* = 123 K
*V* = 556.07 (19) Å^3^	Block, colourless
*Z* = 2	0.24 × 0.16 × 0.16 mm

### Data collection

**Table:**

Bruker Kappa APEXII diffractometer	1370 independent reflections
Radiation source: fine-focus sealed tube	1223 reflections with *I* > 2σ(*I*)
graphite	*R*_int_ = 0.032
φ and ω scans	θ_max_ = 28.2°, θ_min_ = 3.0°
Absorption correction: multi-scan (*SADABS*; Sheldrick, 2004)	*h* = −6→7
*T*_min_ = 0.296, *T*_max_ = 0.390	*k* = −11→10
5032 measured reflections	*l* = −14→14

### Refinement

**Table:**

Refinement on *F*^2^	Primary atom site location: structure-invariant direct methods
Least-squares matrix: full	Secondary atom site location: difference Fourier map
*R*[*F*^2^ > 2σ(*F*^2^)] = 0.025	Hydrogen site location: difference Fourier map
*wR*(*F*^2^) = 0.061	All H-atom parameters refined
*S* = 1.11	*w* = 1/[σ^2^(*F*_o_^2^) + (0.0256*P*)^2^ + 0.322*P*] where *P* = (*F*_o_^2^ + 2*F*_c_^2^)/3
1370 reflections	(Δ/σ)_max_ = 0.001
95 parameters	Δρ_max_ = 0.38 e Å^−^^3^
0 restraints	Δρ_min_ = −0.70 e Å^−^^3^

### Special details

**Table:**

Geometry. All s.u.'s (except the s.u. in the dihedral angle between two l.s. planes) are estimated using the full covariance matrix. The cell s.u.'s are taken into account individually in the estimation of s.u.'s in distances, angles and torsion angles; correlations between s.u.'s in cell parameters are only used when they are defined by crystal symmetry. An approximate (isotropic) treatment of cell s.u.'s is used for estimating s.u.'s involving l.s. planes.
Refinement. Refinement of *F*^2^ against ALL reflections. The weighted *R*-factor *wR* and goodness of fit *S* are based on *F*^2^, conventional *R*-factors *R* are based on *F*, with *F* set to zero for negative *F*^2^. The threshold expression of *F*^2^ > 2σ(*F*^2^) is used only for calculating *R*-factors(gt) *etc*. and is not relevant to the choice of reflections for refinement. *R*-factors based on *F*^2^ are statistically about twice as large as those based on *F*, and *R*- factors based on ALL data will be even larger.

### Fractional atomic coordinates and isotropic or equivalent isotropic displacement parameters (Å^2^)

**Table:**

	*x*	*y*	*z*	*U*_iso_*/*U*_eq_	
N1	0.5241 (3)	0.3260 (2)	0.48286 (16)	0.0111 (4)	
C2	0.6413 (5)	0.1983 (3)	0.4148 (2)	0.0146 (5)	
C3	0.3697 (5)	0.2474 (3)	0.5693 (2)	0.0155 (5)	
C4	0.3920 (4)	0.4292 (3)	0.3948 (2)	0.0120 (4)	
C5	0.2945 (4)	0.5749 (3)	0.4538 (2)	0.0117 (5)	
Br1	0.89763 (4)	0.01387 (3)	0.702200 (19)	0.01391 (10)	
H2A	0.524 (5)	0.137 (3)	0.374 (2)	0.017 (7)*	
H2B	0.720 (5)	0.138 (3)	0.473 (2)	0.013 (7)*	
H2C	0.733 (4)	0.252 (3)	0.356 (2)	0.011 (6)*	
H3A	0.462 (5)	0.186 (3)	0.620 (2)	0.019 (7)*	
H3B	0.292 (5)	0.333 (3)	0.614 (2)	0.020 (7)*	
H3C	0.270 (5)	0.190 (3)	0.524 (3)	0.021 (8)*	
H4A	0.272 (4)	0.370 (3)	0.360 (2)	0.010 (6)*	
H4B	0.491 (5)	0.456 (3)	0.331 (2)	0.010 (6)*	
H5A	0.182 (5)	0.546 (3)	0.519 (2)	0.013 (7)*	
H5B	0.220 (4)	0.637 (3)	0.394 (2)	0.005 (6)*	

### Atomic displacement parameters (Å^2^)

**Table:**

	*U*^11^	*U*^22^	*U*^33^	*U*^12^	*U*^13^	*U*^23^
N1	0.0101 (10)	0.0108 (9)	0.0122 (9)	−0.0002 (7)	0.0004 (7)	−0.0004 (7)
C2	0.0186 (14)	0.0101 (11)	0.0149 (11)	0.0019 (10)	0.0000 (10)	−0.0024 (9)
C3	0.0157 (14)	0.0142 (12)	0.0168 (12)	−0.0011 (10)	0.0034 (10)	0.0033 (9)
C4	0.0128 (12)	0.0105 (10)	0.0122 (10)	0.0004 (9)	−0.0029 (9)	−0.0010 (9)
C5	0.0116 (12)	0.0095 (10)	0.0137 (11)	0.0019 (9)	−0.0034 (9)	−0.0011 (9)
Br1	0.01243 (16)	0.01658 (15)	0.01261 (14)	0.00082 (9)	−0.00030 (9)	−0.00037 (8)

### Geometric parameters (Å, °)

**Table:**

N1—C4	1.505 (3)	C3—H3B	1.01 (3)
N1—C5^i^	1.506 (3)	C3—H3C	0.90 (3)
N1—C2	1.507 (3)	C4—C5	1.524 (3)
N1—C3	1.512 (3)	C4—H4A	0.93 (3)
C2—H2A	0.96 (3)	C4—H4B	0.97 (3)
C2—H2B	0.93 (3)	C5—N1^i^	1.506 (3)
C2—H2C	0.99 (3)	C5—H5A	1.04 (3)
C3—H3A	0.93 (3)	C5—H5B	0.94 (2)
			
C4—N1—C5^i^	108.49 (18)	N1—C3—H3C	105.5 (18)
C4—N1—C2	108.52 (17)	H3A—C3—H3C	113 (2)
C5^i^—N1—C2	107.85 (18)	H3B—C3—H3C	112 (2)
C4—N1—C3	111.58 (19)	N1—C4—C5	112.19 (18)
C5^i^—N1—C3	112.09 (17)	N1—C4—H4A	108.5 (16)
C2—N1—C3	108.19 (18)	C5—C4—H4A	108.6 (16)
N1—C2—H2A	107.1 (16)	N1—C4—H4B	108.0 (16)
N1—C2—H2B	105.1 (16)	C5—C4—H4B	112.5 (15)
H2A—C2—H2B	111 (2)	H4A—C4—H4B	107 (2)
N1—C2—H2C	106.7 (15)	N1^i^—C5—C4	112.47 (19)
H2A—C2—H2C	109 (2)	N1^i^—C5—H5A	104.9 (15)
H2B—C2—H2C	117 (2)	C4—C5—H5A	112.6 (15)
N1—C3—H3A	106.8 (18)	N1^i^—C5—H5B	108.7 (15)
N1—C3—H3B	107.6 (15)	C4—C5—H5B	108.2 (14)
H3A—C3—H3B	112 (2)	H5A—C5—H5B	110 (2)
			
C5^i^—N1—C4—C5	−55.1 (3)	C3—N1—C4—C5	68.8 (2)
C2—N1—C4—C5	−172.1 (2)	N1—C4—C5—N1^i^	57.4 (3)

Symmetry codes: (i) −*x*+1, −*y*+1, −*z*+1.

### Hydrogen-bond geometry (Å, °)

**Table:**

*D*—H···*A*	*D*—H	H···*A*	*D*···*A*	*D*—H···*A*
C2—H2A···Br1^ii^	0.96 (3)	2.88 (4)	3.816 (4)	165 (2)
C2—H2B···Br1	0.93 (3)	2.92 (4)	3.820 (4)	163 (2)
C2—H2C···Br1^iii^	0.98 (3)	2.83 (4)	3.770 (3)	161 (2)
C3—H3B···Br1^iv^	1.00 (3)	2.84 (3)	3.806 (4)	163 (2)
C4—H4A···Br1^v^	0.93 (3)	2.92 (2)	3.566 (2)	127 (2)
C4—H4B···Br1^iii^	0.97 (3)	2.86 (5)	3.787 (2)	159 (2)

Symmetry codes:  (ii) −*x*+1, −*y*, −*z*+1; (iii) *x*, −*y*+1/2, *z*−1/2; (iv) −*x*+1, *y*+1/2, −*z*+3/2; (v) *x*−1, −*y*+1/2, *z*−1/2.
